# In reply: epidemiology of immune-mediated thrombotic thrombocytopenic purpura in Germany

**DOI:** 10.1016/j.rpth.2026.106647

**Published:** 2026-05-30

**Authors:** Anna Y. Scharenberg, Jakob C. Voran, Sarah-Yasmin Thomsen, Anka Pohlmeyer, Victor Walendy, Linus A. Völker, Roland Schmitt, Friedrich A. von Samson-Himmelstjerna, Kevin Schulte, Benedikt Kolbrink

**Affiliations:** 1 Department of Nephrology and Hypertension, University Hospital Schleswig-Holstein, Campus Kiel, Kiel, Germany; 2 Department of Internal Medicine III, Cardiology and Critical Care, University Hospital Schleswig-Holstein, Campus Kiel, Kiel, Germany; 3 DZHK (German Centre for Cardiovascular Research), partner site Hamburg/Kiel/Lübeck, Kiel, Germany; 4 Department of Internal Medicine II, Medical Faculty of the Martin-Luther-University Halle-Wittenberg, Halle, Germany; 5 Department II of Internal Medicine and Center for Molecular Medicine Cologne, Faculty of Medicine and University Hospital Cologne, University of Cologne, Cologne, Germany

Dear Editor,

We thank our colleagues for their important comments [1] on our work on the epidemiology of immune-mediated thrombotic thrombocytopenic purpura (iTTP) in Germany [2]. In response, we would first like to address the methodological concerns raised: The main point is that, under the diagnostic code M31.1 we used—which explicitly includes iTTP in the ICD-10-GM—other thrombotic microangiopathies (TMAs) may also have been coded. This is, in principle, possible, and we attempted as far as possible to exclude all other relevant forms of TMAs from our analyses. To this end, we defined a detailed list of diagnostic codes for other TMAs and associated conditions—including the aforementioned (atypical) hemolytic uremic syndrome—as exclusion criteria for our analyses.

Among these exclusion diagnoses is systemic lupus erythematosus (SLE), as correctly noted. It is well established that a small single-digit percentage of patients with iTTP also have concomitant SLE [3]. Conversely, a larger proportion of patients with SLE develop TMA, of which iTTP is presumed to account for only a minority [4]. Including patients with SLE in our analyses would therefore likely have resulted in the inclusion of a substantially higher number of non-iTTP TMAs than true iTTP cases. Nevertheless, there is evidence suggesting that patients with iTTP and concomitant SLE tend to have better outcomes than those without SLE [5], so that excluding patients with SLE may have led to a slight overestimation of unfavorable outcomes in the overall iTTP cohort.

While we agree that it is widely accepted that acute kidney injury is not very common in iTTP, there are contrary reports and opinions suggesting that it may occur more frequently than previously assumed—even more frequently than observed in our cohort [6]. In our view, a further reason for the comparatively poorer outcomes in our cohort is likely the inclusion of data from nonspecialized centers, where the diagnosis of iTTP may be established later and treatment may be less standardized, as well as a complete capture of all iTTP cases even in larger centers, thereby minimizing selection bias. In addition, based on our data, we cannot exclude the possibility that acute kidney injury and multiorgan failure are not directly caused by iTTP itself but rather by complications of hospitalization and treatment, such as infections, or by preexisting comorbidities (which may be particularly relevant in older patients [7,8]).

Ultimately, in our view, there is no stronger approach for internal validation of the administrative data underlying our analyses than the methods we applied. External validity is supported by the finding that our iTTP cohort is in good agreement with previous reports in terms of incidence rates, demographic characteristics, and the mean number of plasma exchange treatments performed.

We believe that our data provide good reason to assume that certain outcomes of iTTP may have been worse than previously thought. It should be noted, however, that our data largely originate from the pre-caplacizumab era and that outcomes have likely improved substantially following the (now relatively) routine introduction of caplacizumab into iTTP therapy [9].

We fully agree with our colleagues that, in cases of prolonged kidney injury, alternative differential diagnoses beyond iTTP should be strongly considered, and that the indication for a kidney biopsy should be established at a low threshold.
